# Berberine Improves Vascular Dysfunction by Inhibiting Trimethylamine-N-oxide *via* Regulating the Gut Microbiota in Angiotensin II-Induced Hypertensive Mice

**DOI:** 10.3389/fmicb.2022.814855

**Published:** 2022-03-08

**Authors:** Zhichao Wang, Fang Wu, Qianbing Zhou, Yumin Qiu, Jianning Zhang, Qiang Tu, Zhe Zhou, Yijia Shao, Shiyue Xu, Yan Wang, Jun Tao

**Affiliations:** ^1^Department of Hypertension and Vascular Disease, The First Affiliated Hospital, Sun Yat-Sen University, Guangzhou, China; ^2^National-Guangdong Joint Engineering Laboratory for Diagnosis and Treatment of Vascular Disease, The First Affiliated Hospital of Sun Yat-sen University, Guangzhou, China; ^3^Institute of Geriatrics, The First Affiliated Hospital, Sun Yat-Sen University, Guangzhou, China; ^4^Department of Stomatology, The First Affiliated Hospital of Guangdong Pharmaceutical University, Guangzhou, China

**Keywords:** berberine, angiotensin II, hypertension, vascular dysfunction, TMAO, gut microbiota

## Abstract

Berberine (BBR) has been demonstrated to exert cardiovascular protective effects by regulating gut microbiota. However, few studies examine the effect of BBR on the gut microbiota in hypertension. This study aims to investigate the role of BBR in regulating microbial alterations and vascular function in hypertension. C57BL/6 J mice were infused with Ang II (0.8 mg/kg/day) *via* osmotic minipumps and treated with BBR (150 mg/kg/day) or choline (1%) for 4 weeks. Blood pressure was detected by tail-cuff measurement once a week. Abdominal aorta pulse wave velocity (PWV) and endothelium dependent vasodilatation were measured to evaluate vascular function. Vascular remodeling was assessed by histological staining of aortic tissue. The fecal microbiota was profiled using 16S ribosomal DNA (rDNA) sequencing. Plasma trimethylamine (TMA)/trimethylamine-N-oxide (TMAO) and hepatic FMO3 expression were measured. We found that BBR treatment significantly alleviated the elevated blood pressure, vascular dysfunction, and pathological remodeling in Ang II-induced hypertensive mice, while choline treatment aggravated hypertension-related vascular dysfunction. 16S rDNA gene sequencing results showed that BBR treatment altered gut microbiota composition (reduced the Firmicutes/Bacteroidetes (F/B) ratio and increased the abundances of *Lactobacillus*). Moreover, BBR inhibited FMO3 expression and plasma TMA/TMAO production in hypertensive mice. TMAO treatment increased the apoptosis and oxidative stress of human aortic endothelial cells (HAECs) and aggravated Ang II-induced HAECs dysfunction *in vitro*. These results indicate that the protective effect of BBR in hypertension might be attributed (at least partially) to the inhibition of TMAO production *via* regulating the gut microbiota.

## Introduction

Hypertension is the primary risk factor of atherosclerotic cardiovascular disease (ASCVD) and a public health problem worldwide. The development of hypertension is a complex process involving both genetic and environmental risk factors. Benefit from the advancement of sequencing technology in recent years, emerging evidence reveal that the gut microbiota plays an important role in the development and pathogenesis of hypertension (Muralitharan et al., 2020; Avery et al., 2021). Study of both hypertensive patients and rat models demonstrated that gut microbial dysbiosis, including depletion of bacterial richness and increased F/B ratio, was closely associated with hypertension (Yang et al., 2015). In contrast to wild-type C57BL/6 mice, germ-free C57BL/6 mice were protected from Ang II-induced vascular endothelial and smooth muscle dysfunction and blood pressure elevation (Karbach et al., 2016). Furthermore, elevated blood pressure was observed in germ-free C57BL/6 mice transplanted with fecal from hypertensive human donors, which indicates direct effect of gut microbiota on hypertension (Li et al., 2017). Similar results were also reported in germ-free rats (Joe et al., 2020). These findings implicate that the gut microbiota is closely involved in the development of hypertension.

Recent studies suggest that gut microbiota regulates vascular function and blood pressure through altering the production of metabolites (Poll et al., 2020). Gut microbiota-derived metabolite trimethylamine-N-oxide (TMAO) is now recognized as a potentially cardiovascular risk factor (Schiattarella et al., 2017; Dannenberg et al., 2020). Foods enrich in choline, phosphatidylcholine, and carnitine, such as red meat, eggs, and fish, can be digested and converted to trimethylamine (TMA) by gut microbiota, which is further metabolized into TMAO by FMO (flavin monooxygenases), especially by FMO isoform 3 (FMO3) in the liver. Accumulating evidence demonstrate that TMA, TMA-producing bacteria, and TMAO are bound up with hypertension (Hoyles et al., 2018; Zhang et al., 2021). A recent study reported that TMAO induced aortic stiffening and increased systolic blood pressure with aging in mice and humans (Brunt et al., 2021). A meta-analysis of large population showed that there was a significant positive dose-dependent association between circulating TMAO and prevalence of hypertension (Ge et al., 2020). Moreover, gut microbiota metabolite TMAO is showed to facilitate Ang II-induced vasoconstriction and hypertension (Jiang et al., 2021). Hence, TMAO is emerged as a potential therapeutic target for hypertension and vascular dysfunction.

Berberine (BBR), a natural plant alkaloid extracted from *Berberis vulgaris* and *Coptis chinensis* (Huanglian), has been widely used to treat bacterial-caused diarrhea in China (Kong et al., 2020; Yang et al., 2021). Given the poor oral bioavailability and extremely low maximum plasma concentration of BBR, the gut microbiota is considered as a crucial mediator that regulates the pharmacokinetic and biological effects of BBR recently (Lyu et al., 2017; Pan et al., 2019; Habtemariam, 2020). BBR has been reported as a clinically effective remedy for metabolic disorders (including diabetes and dyslipidemia) by altering gut microbiota (Wang et al., 2017; Zhang et al., 2020b). In addition, studies from others and our group showed that BBR treatment improved vascular endothelial functions and blood pressure in hypertension (Wang et al., 2009; Cheng et al., 2013; Zhang et al., 2020a). However, the roles of the gut microbiota and microbiota-derived metabolite in BBR-related beneficial effects on vascular function in hypertension have not yet been unveiled. Therefore, our study aimed to decipher the detailed mechanism by which microbial alterations and microbiota-derived metabolites mediated the therapeutic effect of BBR in hypertension.

## Materials and Methods

### Animal Model

All animal experimental procedures were carried out in strict accordance with the recommendations in the Guide for the Care and Use of Laboratory Animals and were approved by the IEC for Clinical Research and Animal Trials of the First Affiliated Hospital of Sun Yat-sen University (approval number: [2021]048). C57BL/6 J mice (8 weeks, male) were provided from animal research center of the first affiliated hospital of Sun Yat-sen University (Guangzhou, China) and maintained at 22–24°C, 50% relative humidity, with a 12 h light/dark period. C57BL/6 J mice were fed with a standard chow diet and randomly assigned to six groups (*n* = 5 for each group). The mini osmotic pump (MODEL 2004, ALZET, United States) was subcutaneously implanted into mice to deliver either saline or Ang II (0.8 mg/kg/day) for 4 weeks. The 1% choline chloride (Sigma, United States) was administered *via* drinking water for 4 weeks. BBR chloride (Sigma, United States) was given at a dosage of 150 mg/kg/day for 4 weeks by intragastric administration.

### Blood Pressure Measurement

The blood pressure measurement was performed as described previously (Zhang et al., 2020a). Noninvasive detection of blood pressure by tail-cuff measurements was performed once a week at the approximate same time using the Softron BP-2010A tail-cuff system. The duration of experiment was exactly 4 weeks.

### Endothelium Dependent Vasodilatation

Measurement of vessel tension in mice was performed as described previously (Zhang et al., 2020a). Briefly, mice thoracic aorta was cut transversally in 3 mm ring segments. All fat and connective tissues were carefully dissected. Each ring was placed inside a 5-ml heated bath filled with KHS (37°C), gassed with a mixture of 95% O_2_ and 5% CO_2_, and suspended between two L-shaped steel hooks. Vasorelaxation was assayed by organ chamber (DMT 620 M, Denmark). Experiments were initiated by obtaining a reference contractile response to 75 mmol/L KCl. Endothelial dependent vascular relaxation was tested after pre-constriction with 1 uM phenylephrine. Once the vessels reach a steady state contraction, increasing concentrations of acetylcholine (ACh; 10^−9^ to 10^−5^ mol/L) were administered, and the response to each concentration of drug was recorded.

### Abdominal Aorta Pulse Wave Velocity

At the end of the experiment, pulse wave velocity (PWV) in mice from each group were detected by Vevo3100 Ultrasound Machine (FUJIFILM VisualSonics, Canada) as reported previously (Wu et al., 2021). Briefly, mice with abdominal hair removed were anesthetized with 2.5% isoflurane and maintained with 1.5% isoflurane and fixed in a 37°C pre-heated platform with a heart rate of 400–550 bp. MX400 (30 MHz) probes for mice were placed longitudinally below the sternum and xiphoid process to get the full image of the abdominal aorta in both B mode and M mode. The PWV was calculated by the formula: length of abdominal aorta (distal time delay-proximal time delay). Images were taken in both B/M mode, followed by the PWV *via* Vevo® LAB software (FUJIFILM VisualSonics, Canada).

### Aortic Tissue Staining

Descending thoracic aorta at the same location for each sample was fixed in 4% paraformaldehyde. After paraffin embedding, Aortic tissue were cut into 6 μm sections and stained with hematoxylin–eosin (HE), masson trichrome and elastic van gieson (EVG). The thickness and expression of elastic fibers in the aorta were measured by image viewing software (NDP.view2).

### 16S rDNA Sequencing and Microbial Diversity Analysis

To investigate the microbiota community composition after BBR treatment, bacterial 16S rDNA gene sequencing assay was performed as previously described (Shi et al., 2021). Briefly, fresh mice feces were collected and quickly frozen in liquid nitrogen and then stored at −80°C. Microbial DNA was isolated and purified using the HiPure Stool DNA Kits (Magen, Guangzhou, China) according to manufacturer’s protocols. Extracted DNA was used as a template to amplify the bacterial 16S rDNA V3-V4 region with barcode-indexed primers 341F (5’-CCTACGGGNGGCWGCAG-3’) and 806R (5’-GGACTACHVGGGTATCTAAT-3’). The amplicons were normalized, pooled, and sequenced on the Illumina platform by Guangzhou Genedenovo Biotechnology Co. (Guangzhou, China). All chimeric tags were removed using UCHIME algorithm, and finally obtained effective tags for further analysis. The effective tags were clustered into operational taxonomic units (OTUs) with ≥97% similarity threshold. The representative sequence for each OTU was selected and the taxonomic information was annotated using RDP classifier and the SILVA database. The analysis was performed at each taxonomical level (Phylum, Class, Order, Family, and Genus) separately. Both alpha and beta diversity analysis were performed based on the output normalized data.

### Plasma TMA/TMAO Detected by HPLC-MS/MS

The plasma TMA/TMAO was assessed using high-performance liquid chromatography with tandem mass spectrometry (HPLC-MS/MS). Briefly, 10 μl d9-TMA/TMAO (Toronto Research Chemicals Inc., Toronto, Canada) was added to 100 uL plasma; then 300 μl of acetonitrile was added for protein precipitation, vortexed for 1 min, centrifuged at 1,000 rpm, 4°C for 5 min; finally, 200 μl of the remaining supernatant was analyzed after injection into a Waters Atlantis HILIC Silica column.

### Western Blotting

Liver protein was extracted and quantified using RIPA lysis buffer (Beyotime Biotechnology, China) containing protease inhibitors and BCA assay kit (Thermo Fisher Scientific, United States) separately. Protein extracts were separated *via* SDS-PAGE and transferred to polyvinylidene difluoride membranes (Roche, Indianapolis, IN, United States). The primary antibodies used were anti-FMO3 antibody (Proteintech, United States) and anti-GAPDH antibody (Proteintech, United States). Protein bands were visualized using an ECL chemiluminescence system (Thermo Fisher Scientific, United States) analyzed using Image J software.

### Cell Culture and Treatment

Human aortic endothelial cells (HAECs) were purchased from iCell Bioscience Inc. (Shanghai, China). Cells were cultured at 37°C and 5% CO2 in endothelial cell medium ECM (ScienCell, United States) supplemented with 10% fetal bovine serum (Gibco, CA, United States). Cells from passages between 3 and 8 were used in the study. Cells were treated with TMAO (Sigma, United States) and Ang II (Sigma, United States) dissolved in sterile ultrapure water and cultured for 48 h for cell experiment.

### Detection of Apoptosis

Apoptosis rate of HAECs induced by TMAO was detected with Annexin V-FITC/propidium iodide (PI) Apoptosis Detection Kit (BD Biosciences). According to manufacturer’s instructions, the cells were harvested by centrifugation (300 g/5 min), washed with cold PBS and 1× binding buffer. After that, cells were resuspended in 200 μl 1 × binding buffer and incubated with Annexin V-FITC (5 μl) and PI (10 μl) at room temperature in the dark for 20 min. The cells were added 300 μl 1 × binding buffer and tested by flow cytometry (Cytoflex, Beckman Coulter, United States). The apoptosis ratio is equal to the sum of early and late apoptosis ratio.

### ROS and NO Assessment

Total intracellular reactive oxygen species (ROS) and nitric oxide (NO) levels were determined using DCFH-DA (Beyotime, China) and DAF-FM DA (Beyotime, China), respectively. According to the manufacturer’s instructions, HAECs were resuspended with diluted DCFH-DA or DAF-FM DA at a cell concentration of 1–2 × 10^6^/ml, and incubated at 37°C for 20 min. Invert and mix the tube every 3–5 min to make the probe and the cells fully contact, and then wash the cells two times with serum-free cell culture medium to fully remove the fluorescent dyes that have not entered the cells. Fluorescence intensities of cellular ROS and NO were determined by flow cytometry (Cytoflex, Beckman Coulter, United States).

### HAECs Transwell Migration

A total of 3 × 10^4^ HAECs were resuspended in 250 μl serum-free ECM and pipetted into the upper section of a Boyden chamber (Corning, United States). The Boyden chamber was placed in a 24-well culture dish containing 500 μl ECM supplemented with 10% FBS. After 24 h incubation at 37°C, the non-migrating cells in the upper chamber were removed carefully using a cotton swab. The transmigrated cells were fixed with 4% paraformaldehyde for 15 min and 0.3% crystal violet for another 15 min for further enumeration and analysis.

### HAECs Wound Healing

Make a scratch wound across each well of the 6-well plate using a 200 μl pipette tip. Wash three times with serum-free ECM to remove loosely held cells. Then each well was added to low serum ECM medium and incubated for 12 h. The scratch healing was recorded by photographing under an inverted microscope (×200 magnification). Compare 0 and 12 h images and calculate area of the wound closed using image J software.

### HAECs Tube Formation

For tube formation assay, Matrigel matrix (Corning, United States) was warmed up at 4°C overnight. After completely thawed, Matrigel (80 μl) was plated to 96-well plates at the same level to distribute evenly and incubated for 1 h at 37°C. 4 × 10^4^ HAECs were resuspended with complete ECM and placed on the top of the Matrigel. Following incubation at 37°C for 12 h, each well was imaged directly under a microscope, and an average of tubules was counted from three to five random fields.

### Statistical Analysis

All data were presented as means ± standard deviation (SD). Comparisons between two groups were analyzed by two-tailed Student *t* test. Statistical significance of multiple groups was assessed by one-way analysis of variance (ANOVA) followed by Tukey’s test. The linear correlations between variables were analyzed by Pearson’s correlation analysis. Two-tailed values of *p* of less than 0.05 were considered as statistically significant. The analyses were performed using the software GraphPad Prism version 8.0 (GraphPad Software Inc., United States).

## Results

### BBR Lowers Blood Pressure in Ang II-Induced Hypertensive Mice

**Figure 1:**
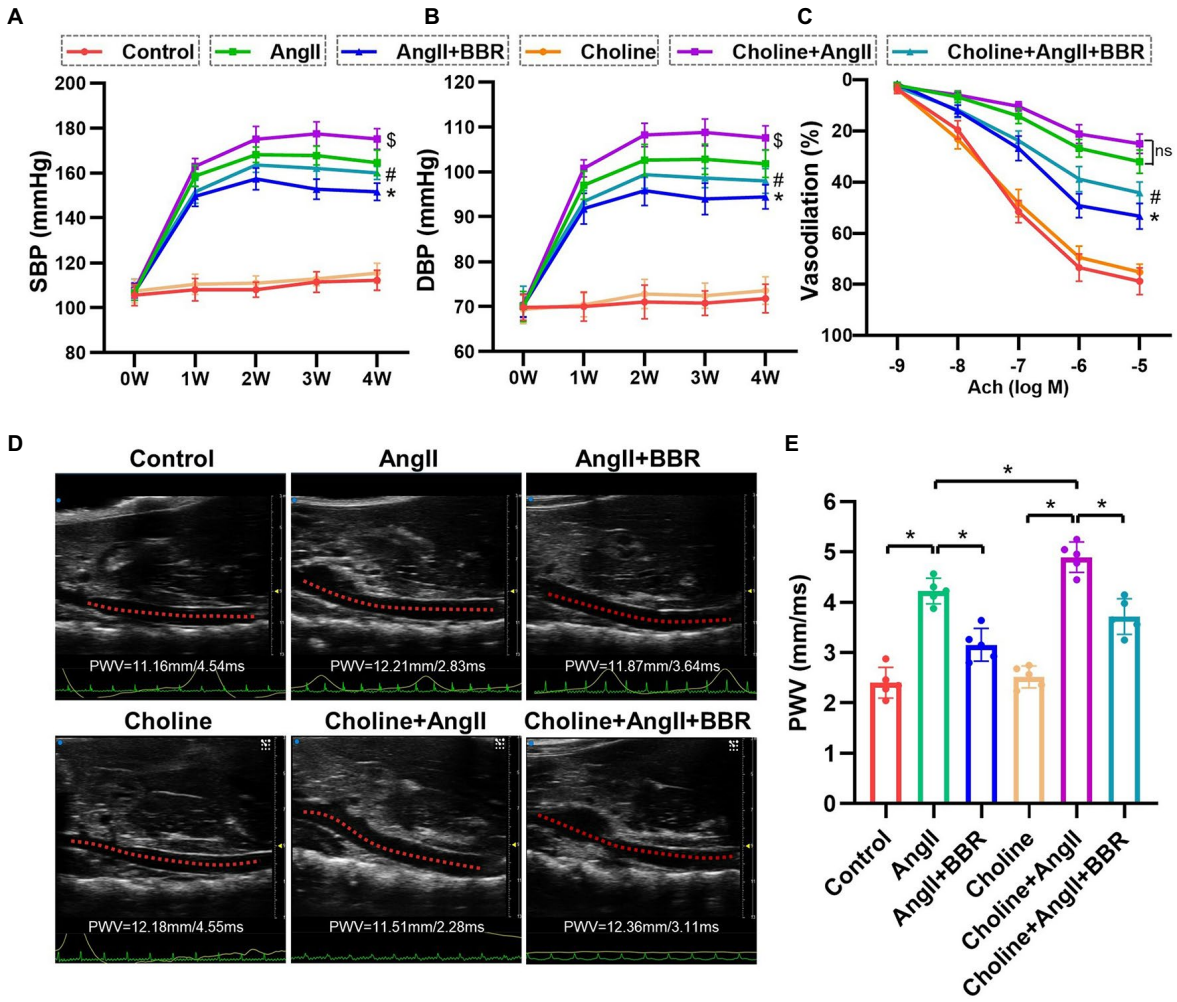
BBR lowers blood pressure and attenuates impaired endothelium dependent vasodilation and elevated PWV in Ang II induced hypertensive mice. **(A)** Systolic blood pressure (SBP), **(B)** diastolic blood pressure (DBP), and endothelium dependent vasodilation **(C)** of mice from each group. **(D)** Representative images of abdominal aorta PWV by ultrasound in each group. **(E)** Quantitative analysis of PWV in each group. *n* = 5 for each group; *p < 0.05 vs. AngII group, ^#^*p* < 0.05 vs. Choline+AngII group, ^$^*p* < 0.05 vs. AngII group.

To investigate the antihypertensive effect of BBR, the blood pressure was measured in Ang II-induced hypertensive mice with or without BBR treatment. Both systolic blood pressure (SBP) and diastolic blood pressure (DBP) were increased in Ang II-infused mice. BBR treatment significantly lowered the blood pressure in Ang II-infused mice (Figures 1A,B). Choline treatment alone had marginal effects on blood pressure. However, hypertension was aggravated by choline in the Ang II-infused mice, which was ameliorated by the supplementation of BBR (Figures 1A,B). Compared with the AngII group, the blood pressure of AngII+BBR group was reduced by 13.0/7.4 mmHg (mean SBP/DBP) after 4 weeks of BBR treatment. Blood pressure in choline+AngII+BBR group was 15.2/9.6 mmHg lower than that in choline+AngII group.

### BBR Ameliorates Vascular Dysfunction and Pathological Remodeling in Ang II-Induced Hypertensive Mice

**Figure 2:**
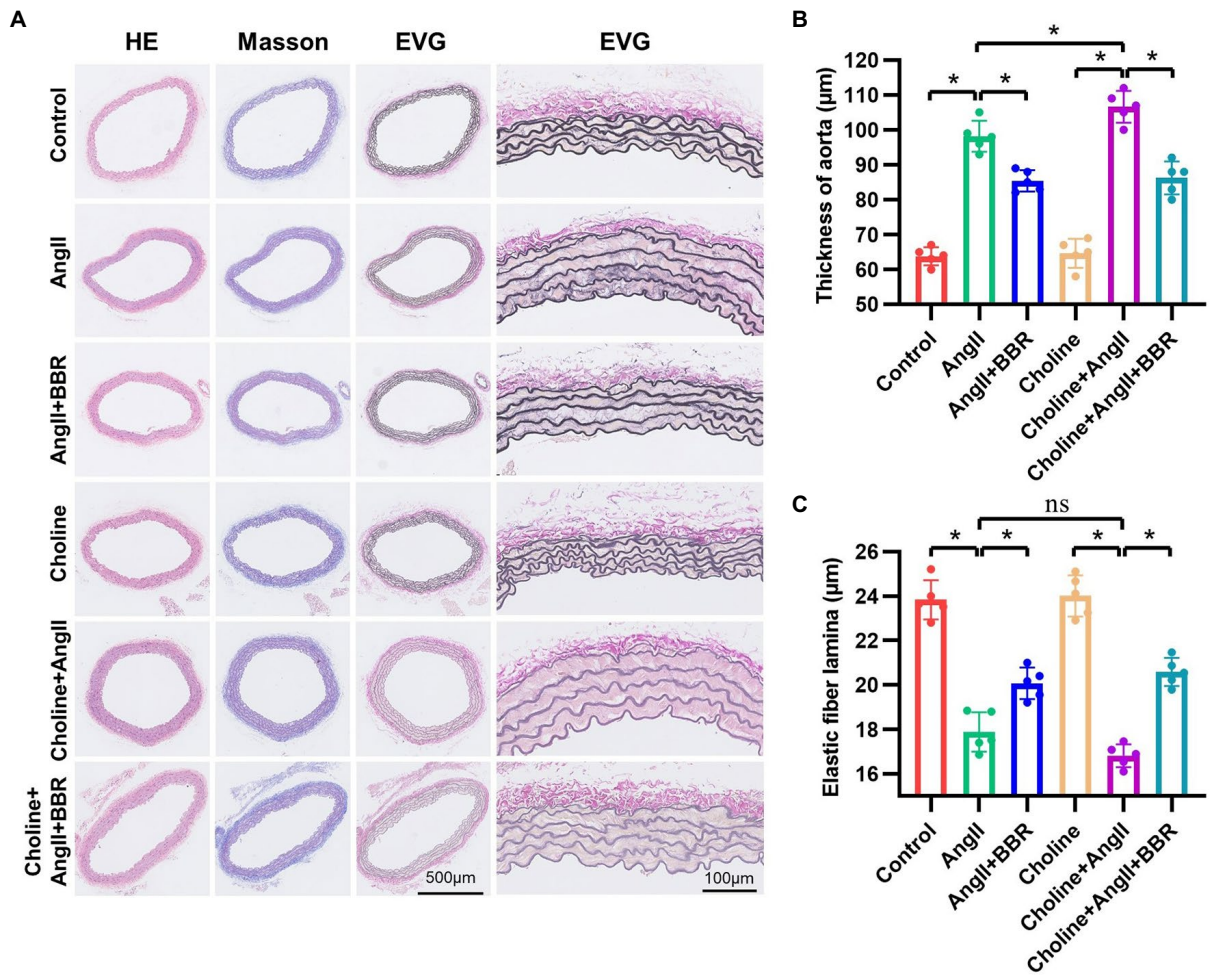
BBR restores the thickness and elastic fiber of aorta in Ang II induced hypertensive mice. **(A)** HE, Masson, and EVG staining of Aortic tissue in each group. Quantitative analysis of media thickness **(B)** and elastic fiber lamina **(C)** of aorta from each group. *n* = 5 for each group; *p < 0.05.

To evaluate endothelial function, ACh-induced endothelium dependent vasodilation of aortic ring was performed. Our results showed that the endothelium-dependent vasodilation was significantly impaired in AngII group compared with the Control group. Importantly, BBR treatment markedly ameliorated the impaired endothelium dependent vasodilation induced by Ang II (Figure 1C). To further assess the effect of BBR on vascular function, we examined the PWV of abdominal aorta by ultrasound (Figure 1D). The PWV of abdominal aorta was increased after 4 weeks of Ang II infusion and significantly attenuated by BBR treatment (Figure 1E). To evaluate the effect of BBR on vascular remodeling induced by Ang II stimulation, the thickness and expression of elastic fibers in the aorta were determined by HE, Masson, and EVG staining (Figure 2A). The media thickness of the aorta was increased by Ang II infusion and decreased by BBR treatment (Figure 2B). BBR treatment also restored Ang II-reduced elastin in aorta, suggesting an improved arterial elasticity (Figure 2C). In addition, choline treatement aggravated Ang II-induced PWV and thickness of aorta, which was ameliorated by the BBR treatment. These results indicated that a protective role of BBR in Ang II-induced vascular dysfunction and pathological remodeling.

### BBR Alters the Gut Microbiota Community Composition

**Figure 3:**
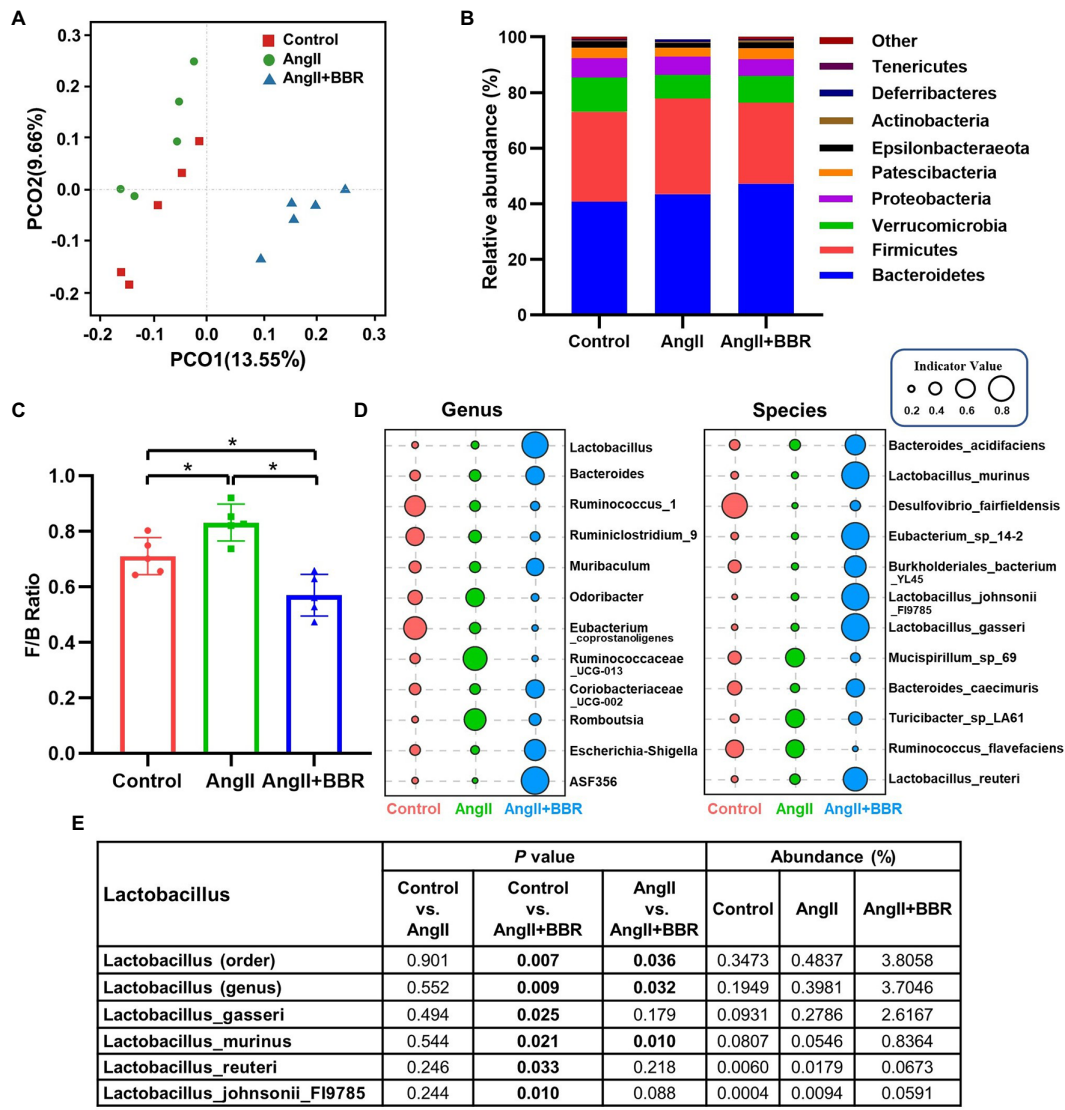
The effect of BBR on gut microbiota community composition. **(A)** Unweighted UniFrac-based PCoA plots illustrating differences in gut microbiota among groups (β-diversity). **(B)** Relative abundance of gut microbiota (phylum level) in each group. **(C)** Firmicutes/Bacteroidetes (F/B) ratio in each group. **(D)** Indicator analysis at genus and species level in each group. **(E)** Abundances of *Lactobacillus* in each group. *n* = 5 for each group; *p < 0.05.

To elucidate whether BBR can remodel the gut microbiota diversity and composition, 16S rDNA gene V3-V4 sequence analysis was performed to analyze 15 fecal samples. The alpha diversity indices (Rank Abundance distribution curve, Chao1, Shannon, and Simpson) were calculated (Supplementary Figure 1). The Shannon and Simpson indices of the gut microbiota community in AngII+BBR group had a higher trend than that of the AngII group, but they were not statistically significant (*p* = 0.095). Based on unweighted UniFrac distance matrices at OTU level, the beta diversity was assessed by principal coordinates analysis (PCoA) and Similarity (ANOSIM) analysis. The PCoA and ANOSIM analysis showed dramatic changes in microbial communities after BBR treatment (AngII+BBR vs. AngII, ANOSIM R = 0.88, *p* = 0.013), indicating a significant shift in the gut bacterial diversity and composition after BBR administration, which Ang II has a relative slight impact on the diversity of the gut microbiota (Control vs. AngII, ANOSIM R = 0.36, *p* = 0.048) (Figure 3A; Supplementary Figure 2). Bacteroidetes, Firmicutes, Verrucomicrobia, and Proteobacteria were the main phyla in all groups at the phylum level, accounting for 92% of the gut microbiota community. A relatively lower abundance of Firmicutes was observed in the AngII+BBR groups (29.12%) than in either Control (32.34%) or AngII (34.45%) groups, while the relative abundance of Bacteroidetes was much higher in the AngII+BBR groups (47.26%) compared to Control (40.77%) or AngII (43.44%) groups (Figure 3B). An increased F/B ratio has been widely considered as a signature of gut dysbiosis. Oral administration of BBR was capable of reducing Firmicutes and increasing Bacteroidetes, which led to the reduction of F/B ratio (Figure 3C). Moreover, we found that BBR administration resulted in an increase in the abundances of Lactobacillus, among which Lactobacillus_gasseri is the most prominent species, followed by Lactobacillus_murinus, Lactobacillus_reuteri and Lactobacillus_johnsonii_FI9785 (Figures 3D,E). In summary, we demonstrated that BBR administration reshaped the microbiota composition in Ang II induced hypertensive mice by regulating the microbial diversity, F/B ratio and the abundances of Lactobacillus.

### BBR Inhibited TMAO Synthesis in Ang II-Induced Hypertensive Mice

**Figure 4:**
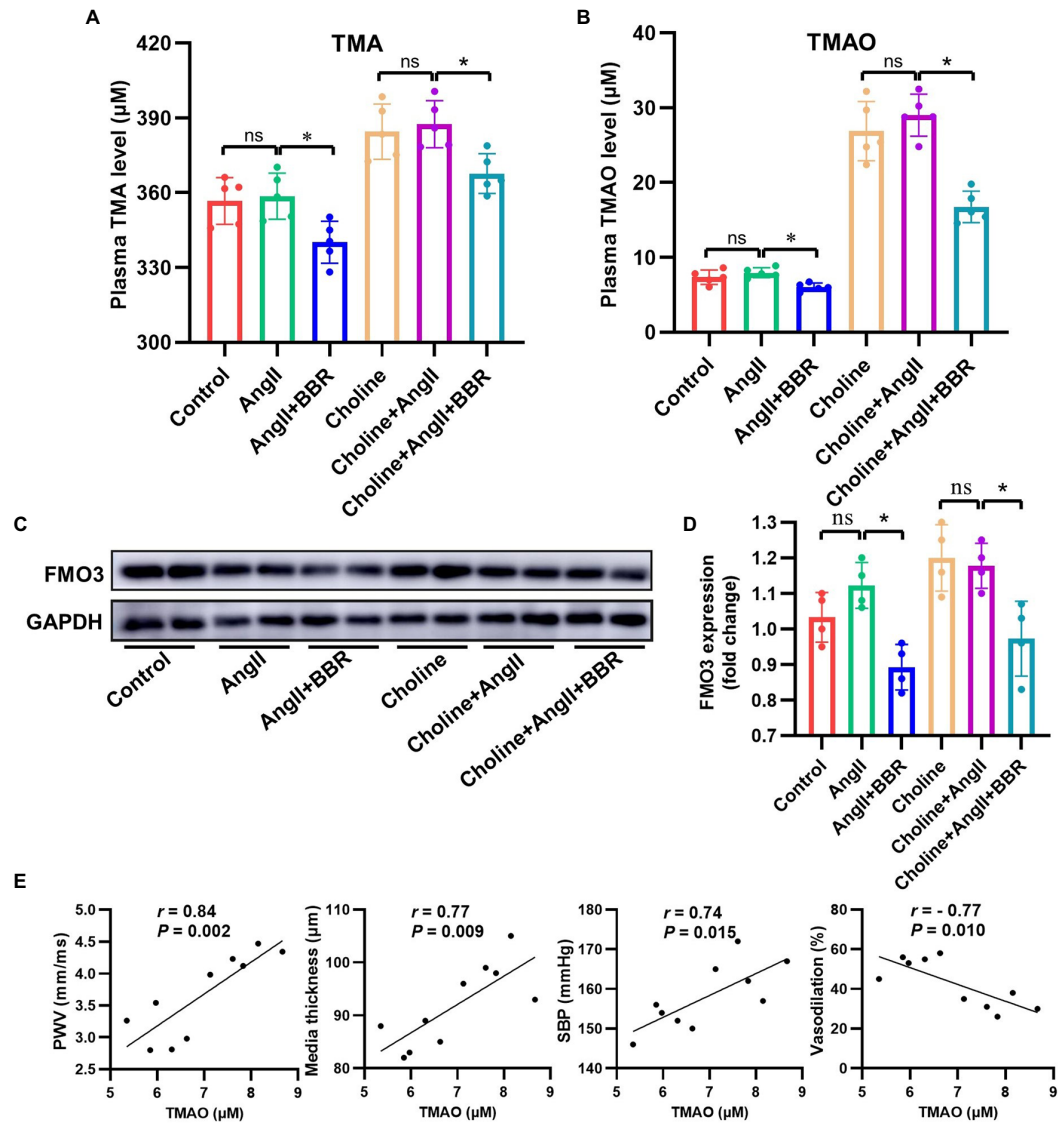
BBR Inhibited TMAO Synthesis in Ang II-Induced Hypertensive Mice. **(A)** Plasma TMA level of each group (*n* = 5). **(B)** Plasma TMAO level of each group (*n* = 5). **(C)** Western blotting detection of FMO3 expression in the liver. **(D)** Statistical analysis of hepatic FMO3 expression of each group (*n* = 4). **(E)** Linear correlation between TMAO level and PWV, media thickness of aorta, SBP and endothelium dependent vasodilation (*n* = 10). *p < 0.05.

To investigate whether BBR affected TMAO synthesis, plasma TMA/TMAO levels were analyzed. We observed a significant lower TMA/TMAO level in the AngII+BBR groups than in the Control group and AngII group. Addition of choline for 4 weeks significantly increased the levels of TMA and TMAO; however, this was reversed by BBR administration (Figures 4A,B). Our results suggested that BBR reduced TMAO synthesis levels in C57BL/6 J mice. TMAO production is dependent on gut microbiota which can metabolize dietary choline to TMA. The TMA is then metabolized by enzymes of the FMO3 in the liver. We therefore determined the expression of FMO3 in the liver. Western blotting of liver proteins showed that treatment with BBR resulted in a significant decrease in protein levels of FMO3 (Figures 4C,D). These results indicated that the BBR-induced reduction in TMAO levels is partly due to its regulation of FMO3 in the liver. Moreover, correlation analysis showed that the TMAO level was positively and linearly associated with the PWV, media thickness of the aorta, and SBP, and negatively associated with the endothelium dependent vasodilation (Figure 4E). These results suggested that BBR-induced reduction of TMAO was associated with the improved vascular function and structure in Ang II-induced hypertensive mice.

### TMAO Promotes the Apoptosis and Oxidative Stress of HAECs

**Figure 5:**
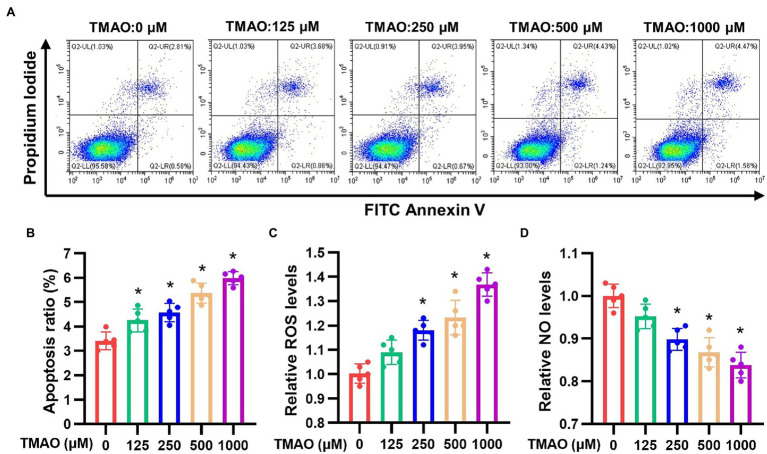
Apoptosis ratio, intracellular ROS and NO levels of HAECs induced by different concentrations of TMAO *in vitro*. **(A)** Representative images of apoptosis ratio. Quantitative analysis of apoptosis ratio **(B)**, relative ROS levels **(C)** and relative NO levels **(D)**. *n* = 5 for each group; *p < 0.05 vs. control group.

We further explored the underlying mechanism of TMAO-mediated vascular endothelial dysfunction. HAECs were treated with various concentrations of TMAO (0, 125, 250, 500, and 1,000 μmol/L) and apoptosis ratio, intracellular ROS and NO were analyzed. Our data showed increase in both apoptosis ratio (Figures 5A,B), intracellular ROS levels (Figure 5C) and a decrease in intracellular NO (Figure 5D) in a dose-dependent manner in HAECs treated with TMAO.

### TMAO Aggravates Ang II-Induced HAECs Dysfunction *in vitro*

**Figure 6:**
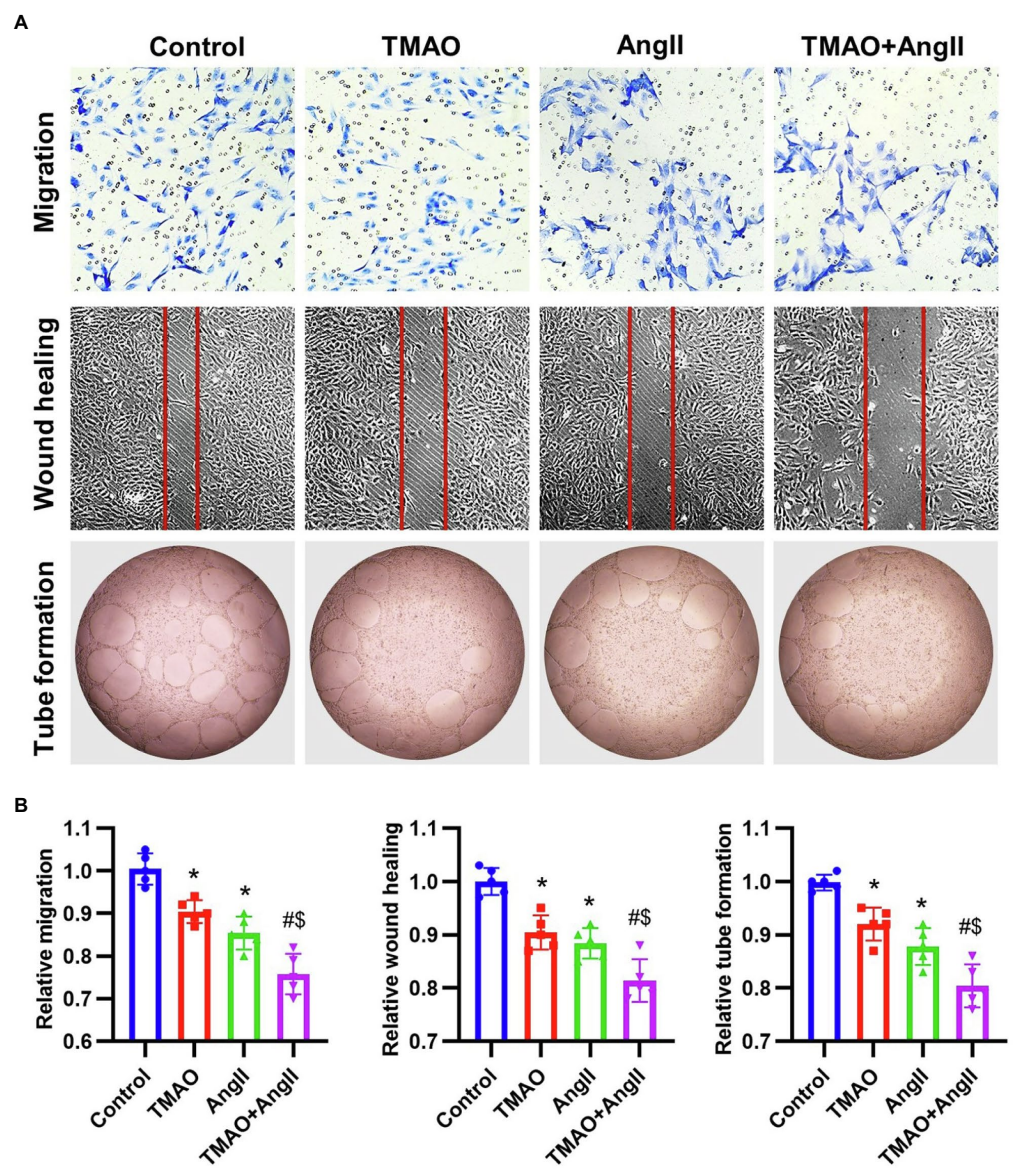
The *in vitro* function of HAECs treated with TMAO (500 μmol/L) and Ang II (2 μmol/L). **(A)** Representative images of *in vitro* function of HAECs (including transwell migration, wound healing and tube formation). **(B)** Quantitative analysis of *in vitro* function of HAECs. *n* = 5 for each group; *p < 0.05 vs. control group, ^#^*p* < 0.05 vs. TMAO group, ^$^*p* < 0.05 vs. AngII group.

To study the effects of TMAO and Ang II on the vascular endothelium, HAECs were treated with TMAO (500 μmol/L) and Ang II (2 μmol/L) and then the *in vitro* functions were evaluated, including transwell migration, wound healing and tube formation (Figure 6A). The *in vitro* functions of HAECs were both significantly impaired under TMAO and Ang II treatment, and TMAO aggravated HAECs dysfunction when combined with Ang II (Figure 6B).

## Discussion

In recent years, the role of BBR in the prevention and treatment of ASCVD is drawing increasing attention (Cai et al., 2021). In the present study, we found that BBR treatment lowered the blood pressure and ameliorated the vascular dysfunction and pathological remodeling in the Ang II-induced mice. We also found that choline aggravates Ang II-induced blood pressure, PWV and thickness of aorta, which was ameliorated by the BBR treatment. More importantly, this beneficial effect of BBR was attributed to the inhibition of TMAO production *via* regulating gut microbiota and FMO3 expression in the liver. Our study demonstrated for the first time that gut microbiota is a critical mediator of BBR-mediated vascular protection in hypertension.

Gut microbiota plays an important role in the initiation, development, and establishment of hypertension (Muralitharan et al., 2020). Gut microbial dysbiosis, decreases in the microbial richness and increases in the F/B ratio, is associated with hypertension (Yang et al., 2015; Adnan et al., 2017; Robles-Vera et al., 2020). Karbach et al. demonstrated that the absence of gut microbiota protects mice from Ang II-induced hypertension, vascular dysfunction (Karbach et al., 2016). In the present study, our data shows significant difference in the composition of microbial communities between AngII+BBR groups and AngII groups by PCoA and ANOSIM analysis. The F/B ratio was considerably increased in the Ang II infused mice compared with controls, which is consistent with that previous report that Ang II-induced gut dysbiosis is characterized by an increase in Firmicutes and a decrease in Bacteroidetes. Studies have shown that BBR can reverse diabetes-related Lactobacillus inhibition (Zhang et al., 2019; Habtemariam, 2020). Recently, a clinic study reported the BBR significantly elevated the relative abundances of Lactobacillus in type 2 diabetes (Zhang et al., 2020b). A systematic review of randomized controlled trials investigating the role of probiotics on high BP showed that Lactobacillus-containing probiotics were effective (Khalesi et al., 2014). Our results show that BBR treatment dramatically increased in the abundances of *Lactobacillus*, especially the Lactobacillus_gasseri, in Ang II-induced hypertensive mice. Therefore, we reasonably infer that BBR may exert cardiovascular protective effects by increasing Lactobacillus level.

As a bioactive metabolite of gut microbiota, TMAO is now recognized as a critical regulator of many cardiovascular diseases. A meta-analysis of a large population showed that individuals with high TMAO were more likely to develop hypertension, and there was a significant positive dose-dependent association between circulating TMAO concentrations and hypertension risk (Ge et al., 2020). Jiang et al. demonstrated that hypertensive patients had higher plasma TMAO than normotensive controls, and TMAO facilitated Ang II-induced vasoconstriction to aggravate Ang II-induced hypertension (Jiang et al., 2021). TMAO also contributes to the age-related endothelial dysfunction *via* oxidative stress in mice and human (Brunt et al., 2020). Thus, TMAO is considered as a novel therapeutic target for hypertension. In consistence with others, our results indicate that TMAO increased the apoptosis ratio and oxidative stress of HAECs and aggravated Ang II-induced HAECs dysfunction *in vitro*, and choline aggravates Ang II-induced blood pressure, PWV and thickness of aorta which was ameliorated by the BBR treatmen *in vivo*. In addition, there was a negative correlation between TMAO and vascular function in AngII and AngII+BBR mice. Studies have demonstrated that oral administration of BBR can effectively reduce the TMAO level in both choline-fed C57BL/6 J and ApoE KO mice, and limit the progression of atherosclerotic plaque area (Shi et al., 2018; Wu et al., 2020; Li et al., 2021). In the present study, we found that BBR inhibited the production of TMA/TMAO in Ang II-induced hypertensive and choline-fed C57BL/6 J mice, and liver protein expression of FMO3 was decreased by BBR treatment, indicating a potential role of the TMA-FMO3-TMAO pathway in the BBR-mediated inhibition of hypertensive development. Moreover, TMAO is closely related to the gut microbiota, such as F/B ratio, lactobacillus abundance. Reports from others showed that the ability of TMA/TMAO production was strong in Firmicutes but very weak in Bacteroidetes (Falony et al., 2015). *Lactobacillus* supplement could reduce plasma TMAO level in mice (Qiu et al., 2018; Liu et al., 2019). Taken together, our data suggest that BBR treatment inhibits TMAO production by modulating gut microbiota and FMO3 expression in Ang II-infused mice, and the reduced TMAO level is associated with BBR-mediated vascular protection.

Collectively, the present study demonstrates for the first time that BBR treatment lowers the blood pressure of hypertensive mice and improves the vascular dysfunction caused by Ang II-induced hypertension. The inhibition of TMAO production *via* restoration of gut microbial dysbiosis attributes to the beneficial effects of BBR administration in Ang II-induced hypertensive mice. Our work may provide new insights into the protective role of BBR in preventing hypertension and ASCVD.

## Data Availability Statement

The original contributions presented in the study are included in the article/Supplementary Material, and further inquiries can be directed to the corresponding authors.

## Ethics Statement

The animal study was reviewed and approved by the IEC for Clinical Research and Animal Trials of the First Affiliated Hospital of Sun Yat-sen University.

## Author Contributions

JT, YW, and SX designed the experiments and performed revisions of the manuscript. ZW, FW, QZ, and YQ conducted the animal and cell experiments and sample analyses. JZ, QT, ZZ, and YS assisted the experiments. ZW, FW, and QZ wrote the draft of the manuscript. All authors contributed to the article and approved the submitted version.

## Funding

This work was financially supported by the National Key R&D Program of China (2020YFC2008005), the National Natural Science Foundation of China (82100451 and 82000461), the Project of Traditional Chinese Medicine Bureau of Guangdong Province of China (20212104), the Natural Science Foundation of Guangdong Province (2020A1515011264), the Science and Technology Planning Project of Guangzhou (202002020030), and the Medical Scientific Research Foundation of Guangdong Province of China (C201903).

## Conflict of Interest

The authors declare that the research was conducted in the absence of any commercial or financial relationships that could be construed as a potential conflict of interest.

## Publisher’s Note

All claims expressed in this article are solely those of the authors and do not necessarily represent those of their affiliated organizations, or those of the publisher, the editors and the reviewers. Any product that may be evaluated in this article, or claim that may be made by its manufacturer, is not guaranteed or endorsed by the publisher.
