# Supplementary material for: Berberine Improves Vascular Dysfunction by Inhibiting Trimethylamine-N-oxide via Regulating the Gut Microbiota in Angiotensin II-Induced Hypertensive Mice
Source: Front Microbiol. 2022 Mar 8;13:814855. doi: 10.3389/fmicb.2022.814855 (PMC8957906; doi:10.3389/fmicb.2022.814855)
Supplement: Supplementary file 1 [file Data_Sheet_1.PDF]

Supplementary Material

Supplementary Figures

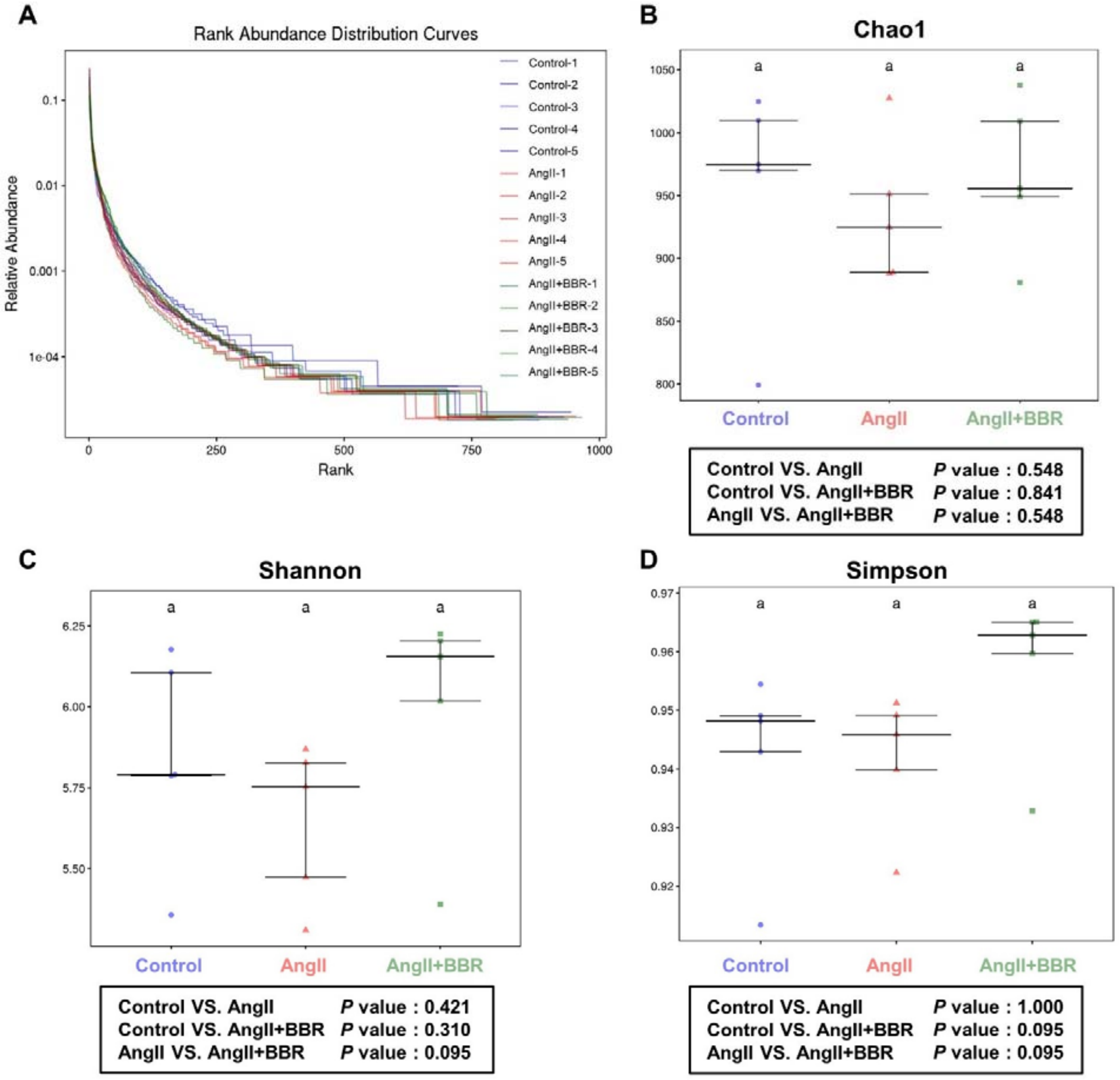

Supplementary Figure 1. Analysis of the alpha diversity of gut microbiota in each group. The richness and diversity were estimated by Rank Abundance Distribution Curve of OTUs (A), the Chao1 index (B), the Shannon index (C) and the Simpson index (D).

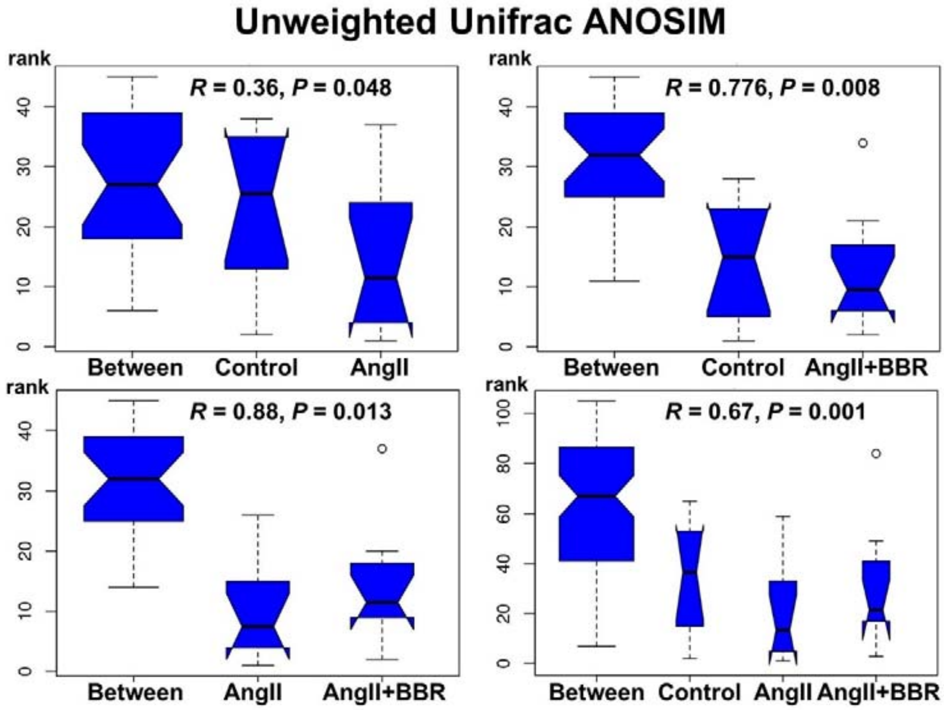

Supplementary Figure 2. Analysis of the beta diversity of gut microbiota in each group by unweighted uniFrac based Similarity (ANOSIM) Analysis. *R* value (0 ~1) represents the degree of difference. Generally, R>0.75: big difference; >0.5: medium difference, >0.25: small difference. *P* value<0.05 indicates statistical significance.
